# Axon morphology of rapid Golgi-stained pyramidal neurons in the prefrontal cortex in schizophrenia

**DOI:** 10.3325/cmj.2020.61.354

**Published:** 2020-08

**Authors:** Ivan Banovac, Dora Sedmak, Martina Rojnić Kuzman, Ana Hladnik, Zdravko Petanjek

**Affiliations:** 1 Department of Anatomy and Clinical Anatomy, Croatian Institute for Brain Research and Center of Excellence for Basic, Clinical and Translational Neuroscience, University of Zagreb School of Medicine, Zagreb, Croatia; 2 University Hospital Centre Zagreb, Zagreb, Croatia; 3 Department of Psychiatry and Psychological Medicine, University of Zagreb School of Medicine, Zagreb, Croatia

## Abstract

**Aim:**

To analyze axon morphology on rapid Golgi impregnated pyramidal neurons in the dorsolateral prefrontal cortex in schizophrenia.

**Methods:**

Postmortem brain tissue from five subjects diagnosed with schizophrenia and five control subjects without neuropathological findings was processed with the rapid Golgi method. Layer III and layer V pyramidal neurons from Brodmann area 9 were chosen in each brain for reconstruction with Neurolucida software. The axons and cell bodies of 136 neurons from subjects with schizophrenia and of 165 neurons from control subjects were traced. The data obtained by quantitative analysis were compared between the schizophrenia and control group with the *t* test.

**Results:**

Axon impregnation length was consistently greater in the schizophrenia group. The axon main trunk length was significantly greater in the schizophrenia than in the control group (93.7 ± 36.6 μm vs 49.8 ± 9.9 μm, *P* = 0.032). Furthermore, in the schizophrenia group more axons had visibly stained collaterals (14.7% vs 5.5%).

**Conclusion:**

Axon rapid Golgi impregnation stops at the beginning of the myelin sheath. The increased axonal staining in the schizophrenia group could, therefore, be explained by reduced axon myelination. Such a decrease in axon myelination is in line with both the disconnection hypothesis and the two-hit model of schizophrenia as a neurodevelopmental disease. Our results support that the cortical circuitry disorganization in schizophrenia might be caused by functional alterations of two major classes of principal neurons due to altered oligodendrocyte development.

Schizophrenia is a psychiatric disorder that manifests as various symptoms: positive (hallucinations and delusions), negative (flattened affect and apathy), and cognitive (memory, attention, and reasoning deficits) (1). Its etiology and pathogenesis remain unresolved. Although it is generally accepted that a combination of genetic and environmental factors is necessary for the clinical manifestation of schizophrenia, the exact mechanisms are still not completely understood. Several hypotheses on the origins of schizophrenia have been proposed. Most of these are not necessarily mutually exclusive. They are based on either altered levels of different neurotransmitters (such as dopamine, GABA, and glutamate) or reduced cortical synaptic connectivity (1). The dopamine hypothesis suggests that psychotic symptoms in schizophrenia are a result of excessive dopamine D2 receptor activation and is supported by the effectiveness of anti-psychotic drugs that block D2 receptors (2-4). The glutamate hypothesis suggests that the etiology of both positive and negative symptoms could be explained by the dysfunction of N-methyl-D-aspartate (NMDA) glutamate receptors, particularly in the prefrontal cortex (PFC) – this is supported by the fact that ketamine, which blocks NMDA receptors, causes schizophrenia-like symptoms and is, therefore, used as a pharmacological model for schizophrenia (5,6). The GABA hypothesis explains the pathogenesis of schizophrenia by alterations in the GABAergic network, such as altered GABA synthesis and re-uptake in the dorsolateral prefrontal cortex (dlPFC) (7,8).

The disconnection hypothesis is of particular interest and is in line with the two-hit model of schizophrenia as a neurodevelopmental disease (1,9,10). It supposes reduced or dysfunctional synaptic connectivity between different cortical areas, particularly the mesocortical pathway involving the PFC. Functional connectivity might be greatly affected by structural alterations of specific neuron classes that have a major role in the integration of cortico-cortical and cortico-subcortical networks, such as layer IIIC and layer V neurons in the dlPFC. Layer IIIC pyramidal neurons have long contralateral and ipsilateral associative (cortico-cortical) projections. Layer V pyramidal neurons have projections to the basal nuclei and are the principal cells of the associative cortex – basal nuclei circuit (11,12). Postmortem studies have shown that schizophrenia patients have reduced soma size, spine densities, and smaller dendritic arbors on pyramidal cells in the PFC (13-15). A decrease in synaptic protein messengers and synaptophysin in the dlPFC was also found (1). Interestingly, there are no studies focusing on axonal properties and morphology in schizophrenia.

Recent studies have revealed that abnormalities in oligodendrocytes lead to altered myelination in schizophrenia (16-23). Myelination is a complex process that includes proliferation, migration, and differentiation of oligodendrocyte precursor cells, membrane outgrowth, axonal wrapping and myelin compaction with node formation (24). Normally, myelin is dynamically regulated by experience during development and in adulthood, having a role in brain plasticity (24). Defects in the myelination process in schizophrenia are likely to affect axon conductivity and contribute to the disconnection hypothesis.

It is well-established that the degree of myelination affects axon impregnation in Golgi staining (25,26). This means that it is possible to indirectly assess myelination by evaluating the axon impregnation in Golgi staining. The rapid Golgi method homogeneously stains neural cell structures, including cell bodies, dendrites, and axons, enabling clear visualization of morphological details. Rapid Golgi stains the cerebral cortex more clearly than other parts of the brain and is particularly well suited for evaluating axon staining and for observing detailed anatomical morphology of neurons (27). The aim of this study is, therefore, to analyze axon morphology in schizophrenia in the dlPFC on rapid Golgi slides.

## MATERIAL AND METHODS

### Brain tissue samples

Quantitative Golgi analysis was performed on the brain samples of ten human subjects aged from 30 to 86 years with a postmortem delay of 6 to 24 hours (Table 1).

**Table 1:** Characteristics of brain tissue samples of controls and subjects with schizophrenia

Sample	Sex	Age (years)	Postmortem delay (h)	Cause of death	Neuropathology	Number of analyzed neurons
CO171	M	86	6	multiple trauma	none	30
CO180	M	30	11	multiple trauma	none	38
CO211	M	58	14.5	sudden cardiac death	none	32
CO215	F	52	24	multiple trauma	none	30
CO246	F	62	11	pneumonia	none	35
CO193	F	53	18	suicide (hanging)	schizophrenia	31
CO195	F	39	22	sudden cardiac death	schizophrenia	23
CO249	M	64	10.5	sudden cardiac death	schizophrenia	19
CO250	M	60	17	sudden cardiac death	schizophrenia	30
CO253	M	55	14.5	sudden cardiac death	schizophrenia	33

Five subjects had no medical history of neurological or psychiatric disorders and no neuropathological deviations in the brain on autopsy. Another five samples were taken from patients with diagnosed schizophrenia during life according to DSM III and ICD-9 criteria. Relevant medical history was obtained from both autopsy reports and medical records. All analyzed subjects died without preagonal state, and the postmortem delay represents the actual interval of neuron death. The brain tissue is a part of the Zagreb Neuroembryological Collection (28,29). It was obtained with the approval of the Ethics Committee of Zagreb University School of Medicine (380-59-10106-14-55/152). The information on the subject’s identity and history is stored in secure records, and the brain tissue is given a code indicating only the subject's age. The analysis was performed during 2018.

The brain tissue was cut into blocks following Talairach coordinates (30). Tissue blocks of the dlPFC from the right hemisphere containing the superior frontal gyrus (Brodmann area 9) were selected for rapid Golgi staining (31). Brodmann area 9 was delineated on neighboring Nissl slides according to relevant literature (32). Cortical layers were also distinguished using neighboring Nissl slides.

### Golgi staining

The tissue was processed with the classical chrome-osmium rapid Golgi staining method (11,33-36). It was placed in 4% paraformaldehyde for 12-18 h and afterwards immersed in rapid Golgi solution consisting of 0.3% osmium tetroxide and 3% potassium dichromate for seven days. The solution was replaced with 1% silver nitrate, in which the tissue was immersed for two days. These steps were performed in a darkened room. The tissue blocks were then dehydrated in an ethanol cascade (70%, 96%, absolute ethanol) and put in alcohol-ether (1:1). The tissue segments were rapidly embedded in celloidin and serially cut on a microtome into coronal slides at a thickness of 200 μm. They were briefly dehydrated (50%, 70%, 96%, ethanol, butanol-ethanol) and placed into Histoclear and mounted with Histomount (National Diagnostics, Atlanta, GA, USA).

The success of Golgi impregnation was determined in accordance with relevant literature (12,34,37,38). No staining artifacts due to postmortem delay were detected.

### Neurolucida three-dimensional reconstructions

Pyramidal neurons with large and medium-sized cell bodies (average cell body area was 347 μm^2^) from layers III and V of Brodmann area 9 were selected for three-dimensional reconstruction with Neurolucida 4 software (MBF Bioscience, Williston, Vermont, USA) using a 60 × air objective of an Olympus BX50 microscope connected to a Hitachi 3CCD color video camera HV-C20M. The neurons were selected for reconstruction as follows: slides of brain tissue from Brodmann area 9 were numbered and randomly chosen for analysis. Subsequently, pyramidal neurons from a region of 3 mm pial length that encompassed all cortical layers meeting the following criteria were selected: the neurons had to be positioned in the middle of the section and the axons should not be cut on the slide edge. Another criterion was that the neurons had fully impregnated somata as well as four or more basal dendrites with at least third-order dendritic branching, in order to ensure adequate impregnation (39). Fusiform-modified pyramidal neurons, typical for layer VI, were not included in the analysis. A total of 165 neurons from control brains (the number of analyzed neurons per subject: 30, 38, 30, 32, and 35) and 136 neurons from brains with schizophrenia (the number of analyzed neurons per subject: 31, 23, 19, 30, and 33) were selected for reconstruction (Table 1). Of the analyzed neurons, 75%-80% were from layer III and 20%-25% were from layer V.

The cell body and axons were traced separately. For the axon, the shaft branch order was applied. The main trunk of the axon directed to the white matter was designated as first order, while the collateral branches were designated as second order segments. For potential branches of second order segments, the centrifugal branch order was applied. The three-dimensional reconstructions were analyzed with Neurolucida Explorer 4 (MBF Bioscience). The *Branched Structure Analysis* function was used to obtain relevant data for the cell bodies and axons (36).

For cell bodies, the parameter *Area* was analyzed. *Area* refers to the surface within the boundary of the cell body in μm^2^. For axons, the parameters *Length, Surface, Volume, Tortuosity, Base Diameter,* and *Average Diameter* were analyzed. *Length* refers to the total length of line segments used to trace a stained axon segment. *Surface* and *Volume* refer to the total surface area and total volume of a stained axon segment, respectively, and are computed by modeling the pieces of the segment as truncated circular cones. *Tortuosity* is the ratio between the length of a stained axon segment and the distance (straight line) between the endpoints of the segment. A straight axon segment would have tortuosity of 1, and tortuosity increases with the complexity of the path of the axon segment. *Base Diameter* represents the diameter of the axon segment at the start of the segment, while *Average Diameter* is a length-weighted mean of the diameter along the stained axon segment.

The length of the axon main trunk and of the axon collaterals were analyzed separately as “axon main trunk length” and “axon collateral length.” “Axon main trunk length” refers to the average stained length of the main trunk of the axon, ie, the average length of first order axon segments. The parameter “axon collateral length” refers to the average stained length of the axon collaterals, ie, the average length of second order axon segments. *Surface, Volume*, *Tortuosity, Base Diameter,* and *Average Diameter* were evaluated only for the axon main trunk, ie, for first order axon segments (parameters: “axon main trunk surface,” “axon main trunk volume,” “tortuosity of axon main trunk,” “base diameter of axon main trunk,” and “average diameter of axon main trunk”).

### Quantitative data analysis

Quantitative data analysis was performed with GraphPad Prism, version 8.3.0 (GraphPad Software, La Jolla, CA, USA) and SPSS, version 26 (IBM Corp., Armonk, NY, USA).

To ensure that the schizophrenia and control groups differ in as few parameters as possible (except in neuropathology), the two groups were compared for age and post-mortem time using the Mann-Whitney test. *P* < 0.05 was considered statistically significant. Both groups contained three male and two female subjects (Table 1), which eliminated possible bias regarding sex.

The analyzed parameters (axon main trunk length, axon main trunk surface, axon main trunk volume, tortuosity of axon main trunk, base diameter of axon main trunk, average diameter of axon main trunk, and axon collateral length) pertaining to neurons from each brain were shown to likely be sampled from a log-normal distribution. The distribution of the parameters was evaluated using GraphPad Prism’s in-built software, which runs four normality and four log-normality tests. The software fits a normal or log-normal distribution using the maximum likelihood method and compares the two likelihoods. Since the logarithmic distribution is characterized by the presence of extremely high values, to which the arithmetic mean is very sensitive, a more appropriate measure of central tendency to describe such data sets is the geometric mean. Therefore, the data for individual brains as well as for all pooled data are presented as geometric mean ×  ÷ geometric standard deviation factor (GSDF) (40).

For every analyzed parameter pertaining to the axon main trunk, the geometric mean was calculated for each individual brain. These geometric means were used to calculate the means and standard deviations for the schizophrenia and control groups. For parameters pertaining to axon collaterals, only pooled data are presented.

For comparison between the schizophrenia and control groups, *t*-test was used. *P* < 0.05 was considered statistically significant.

Since the data are obtained from a hierarchical model, in order to verify the validity of the comparison between the groups, we also addressed different levels of variability by modeling the data using a generalized linear mixed model (GLMM). The advantage of a GLMM is that it can account for non-normal distributions as well as for a multilevel (hierarchical) data structure with both random and fixed effects (41-43). In particular, the analysis addressed the variability of data within a subject, between subjects, and between groups. Neuropathology (presence or absence of schizophrenia) was modeled as a fixed effect, while the subjects and neurons within the subjects were modeled as random effects. The analyzed parameters defined beforehand were modeled as dependent variables (“target” in SPSS). The estimated marginal means were computed based on the original scale of the target.

Correlation between several possible confounding factors (cell body *Area*, age, and postmortem delay) and “axon main trunk length” was determined. Spearman’s rank correlation coefficient ρ was used as a measure for the strength of the association (44). The *t*-test was used to determine whether the observed correlation coefficient could result from random sampling, with *P* < 0.05 being considered statistically significant.

## RESULTS

**Figure 1:**
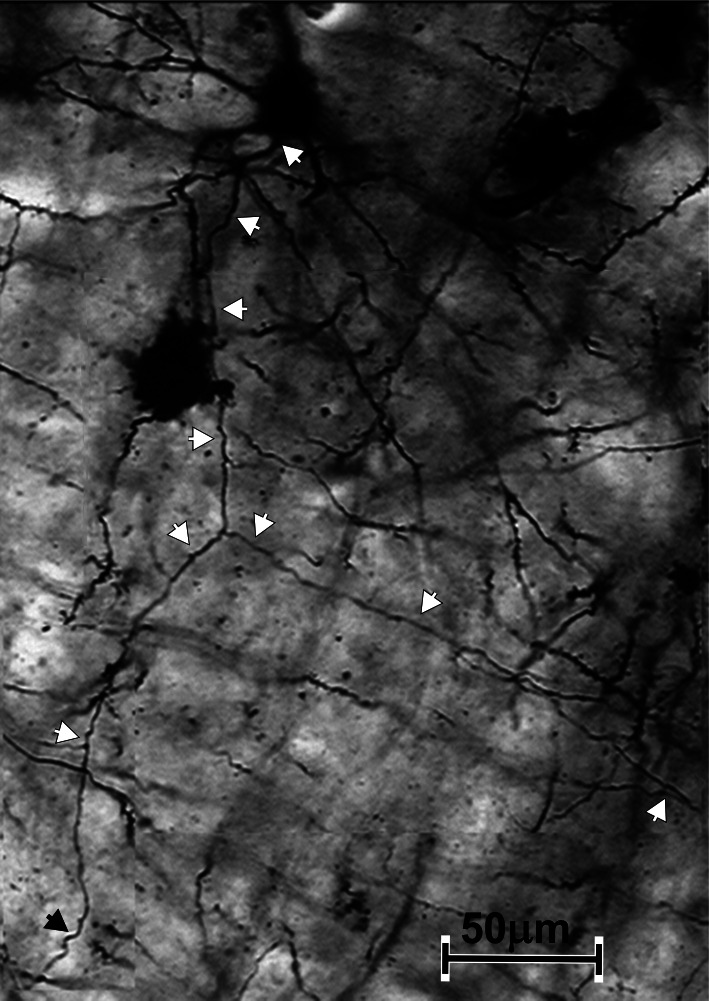
High-power microphotograph of a rapid Golgi impregnated slide from a schizophrenia subject (CO195). The figure is a composition of several microphotographs of the same slide taken at different section depths. The arrowheads indicate the impregnated axon.

**Figure 2:**
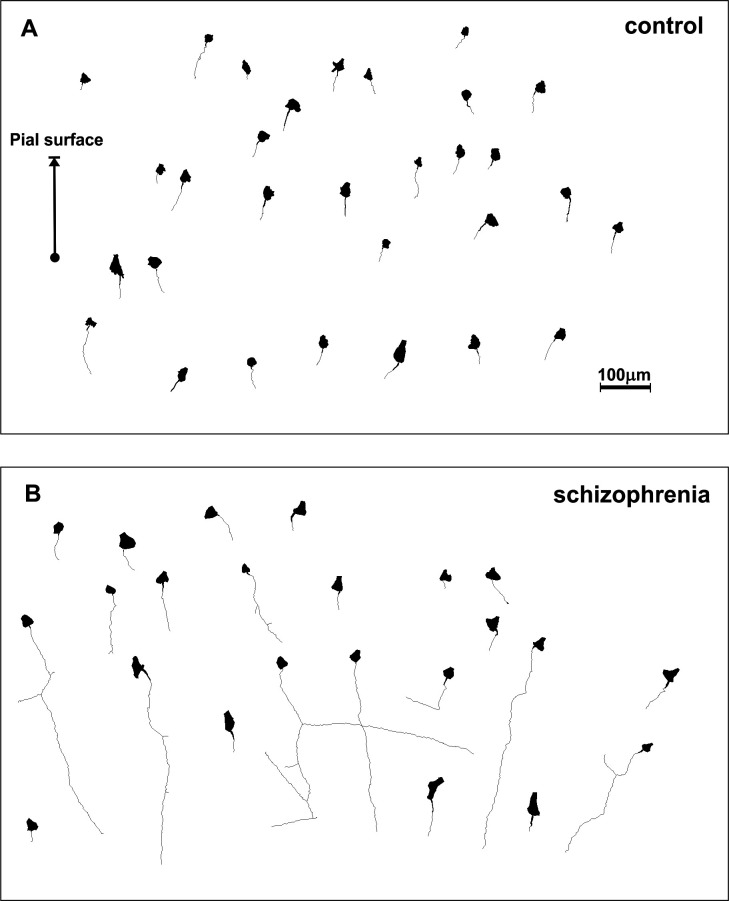
Neurolucida three-dimensional reconstruction of rapid Golgi impregnated axons and neuron cell bodies. (**A**) CO180 from the control group and (**B**) CO195 from the schizophrenia group.

A qualitative evaluation of the rapid Golgi slides revealed an increase in the length of axon impregnation in the schizophrenia group (Figure 1 and Figure 2). This observation was confirmed by quantitative analysis of the Neurolucida three-dimensional reconstructions. No qualitative or quantitative differences were observed between layer III and layer V neurons and, therefore, neurons from both layers were analyzed jointly.

### Axon main trunk

**Figure 3:**
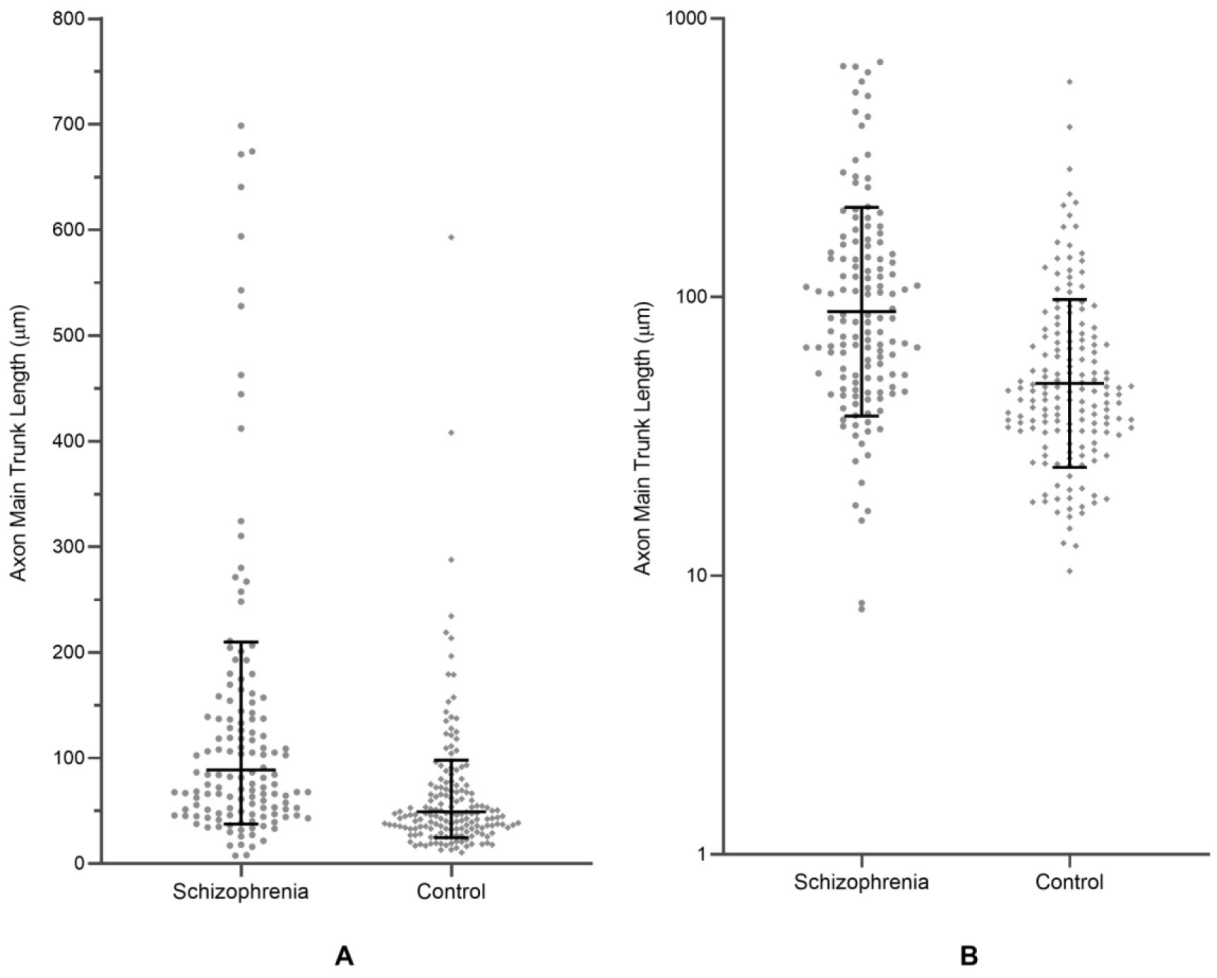
Pooled data showing the comparison of axon main trunk length between the schizophrenia and control group. The black lines and error bars denote the geometric means and geometric standard deviation factors. (**A**) Individual data points plotted on a linear axis. (**B**) Individual data points plotted on a logarithmic axis.

**Figure 4:**
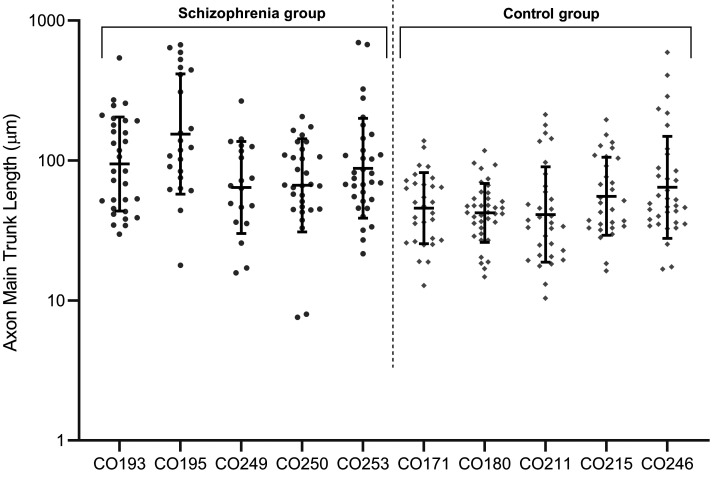
Comparison of axon main trunk length between the schizophrenia and control group. The individual values are plotted on a logarithmic axis. The black lines and error bars denote the geometric means and geometric standard deviation factors.

**Figure 5:**
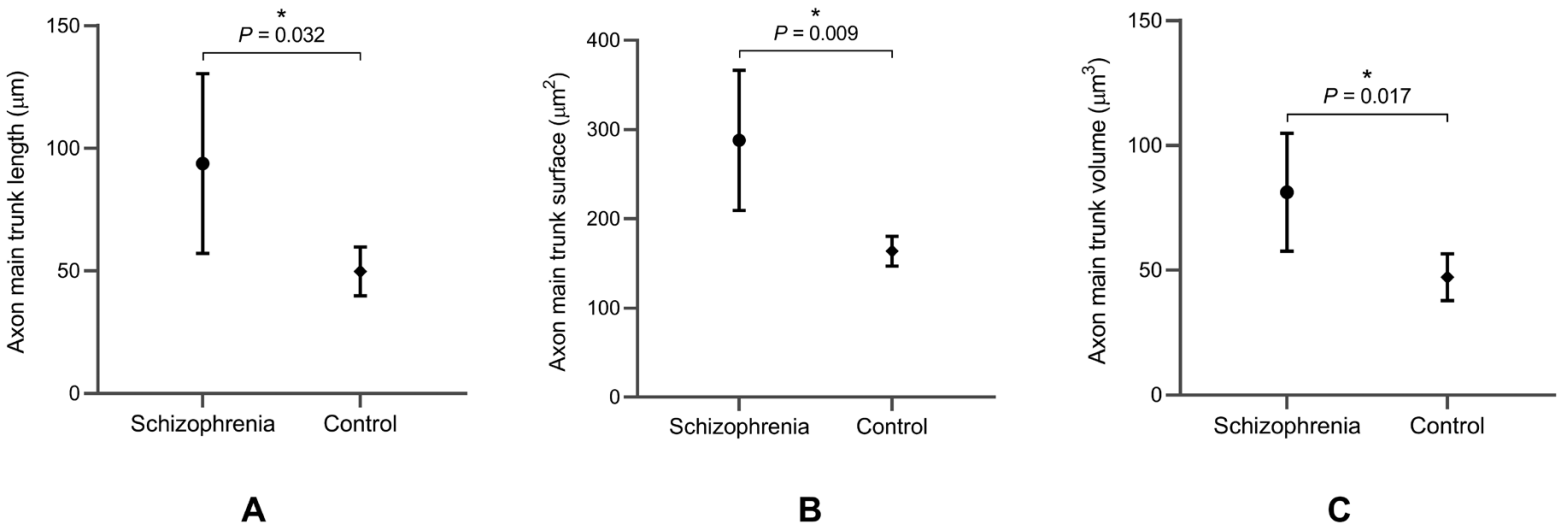
Comparison of (**A**) axon main trunk length, (**B**) axon main trunk surface, and (**C**) axon main trunk volume between the schizophrenia and control group. The black circles and rhombs denote the arithmetic means of the schizophrenia and control group, respectively (derived from the geometric means of individual brains within each group). The error bars denote the standard deviation. Significant differences are marked by asterisks.

**Figure 6:**
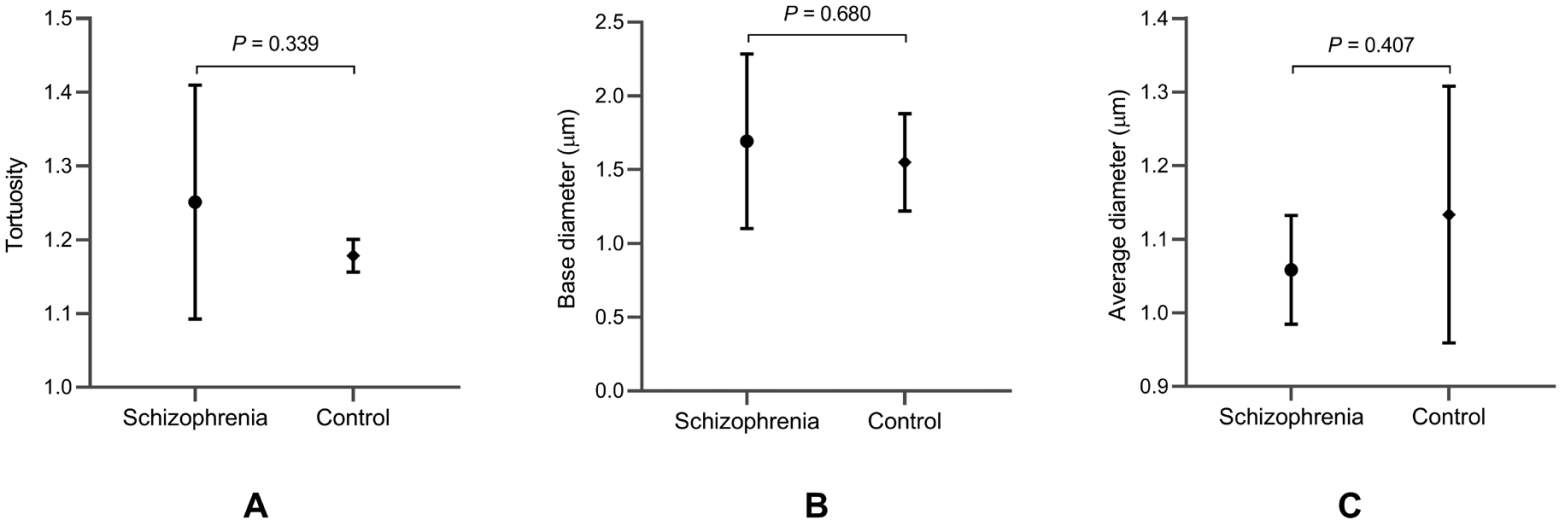
Comparison of (**A**) tortuosity, (**B**) base diameter, and (**C**) average diameter of the axon main trunk between the schizophrenia and control group. The black circles and rhombs denote the arithmetic means of the schizophrenia and control group, respectively (derived from the geometric means of individual brains within each group). The error bars denote the standard deviation.

The analysis of pooled data for the axon main trunk length showed that the axon main trunk length was substantially greater in the schizophrenia (88.7 μm ×  ÷ 2.4) than in the control group (49.0 μm ×  ÷ 2.0) (Figure 3). This was also observed in the analysis of average values per subject – axons from the schizophrenia group had significantly longer stained segments (93.7 ± 36.6 μm) than those from the control group (49.8 ± 9.9 μm, *P* = 0.032) (Figure 4 and Figure 5A). The axon main trunk surface (287.9 ± 78.6 μm^2^ vs 163.7 ± 16.7 μm^2^, *P* = 0.009, Figure 5B) and main trunk volume (81.2 ± 23.7 μm^3^ vs 47.2 ± 9.4 μm^3^, *P* = 0.017, Figure 5C) were also significantly greater in the schizophrenia group than in the control group. There were no significant differences in tortuosity, base diameter, and average diameter of the axon main trunk between the schizophrenia and control group (Figure 6).

### Axon collaterals

The axon collateral length was greater in the schizophrenia group (33.1 μm ×  ÷ 3.0) than in the control group (22.3 μm ×  ÷ 2.7). Furthermore, the neurons in the schizophrenia group had an average number of collaterals per axon of 0.22, compared with 0.07 in the control group. More axons had visibly stained collaterals in the schizophrenia group (14.7%) than in the control group (5.5%).

### Results of generalized linear mixed model analysis

The GLMM analysis revealed that for all the analyzed parameters, the random effects were not significant. The fixed effect (neuropathology) was significant for axon main trunk length, axon main trunk surface, and axon main trunk volume, while it was not significant for tortuosity, base diameter, and average diameter (Table 2).

**Table 2:** Results of generalized linear mixed model analysis

Target (analyzed parameter, ie, dependent variable)	Fixed coefficient – neuropathology = schizophrenia			Fixed coefficient – neuropathology = control			Fixed effect (neuropathology) – *P*	1st random effect (subjects) – *P*	2nd random effect (neurons) – *P*
	estimated mean	standard error	95% confidence interval	estimated mean	standard error	95% confidence interval			
**Axon main trunk length**	123.277	16.767	[92.560, 156.275]	63.962	14.532	[35.365, 90.280]	0.008	0.240	0.923
**Axon main trunk surface**	360.382	27.113	[307.025, 413.739]	196.182	18.064	[160.632, 231.732]	<0.001	0.601	0.884
**Axon main trunk volume**	101.416	8.786	[84.126, 118.707]	57.168	7.0184	[43.356, 70.980]	<0.001	0.330	0.948
**Tortuosity**	1.259	0.053	[1.155, 1.362]	1.185	0.052	[1.082, 1.287]	0.316	0.069	0.772
**Base diameter**	1.793	0.212	[1.376, 2.210]	1.558	0.208	[1.148, 1.968]	0.430	0.074	0.902
**Average diameter**	1.103	0.082	[0.942, 1.264]	1.084	0.081	[0.924, 1.243]	0.865	0.070	0.346

### Analysis of confounding factors

**Figure 7:**
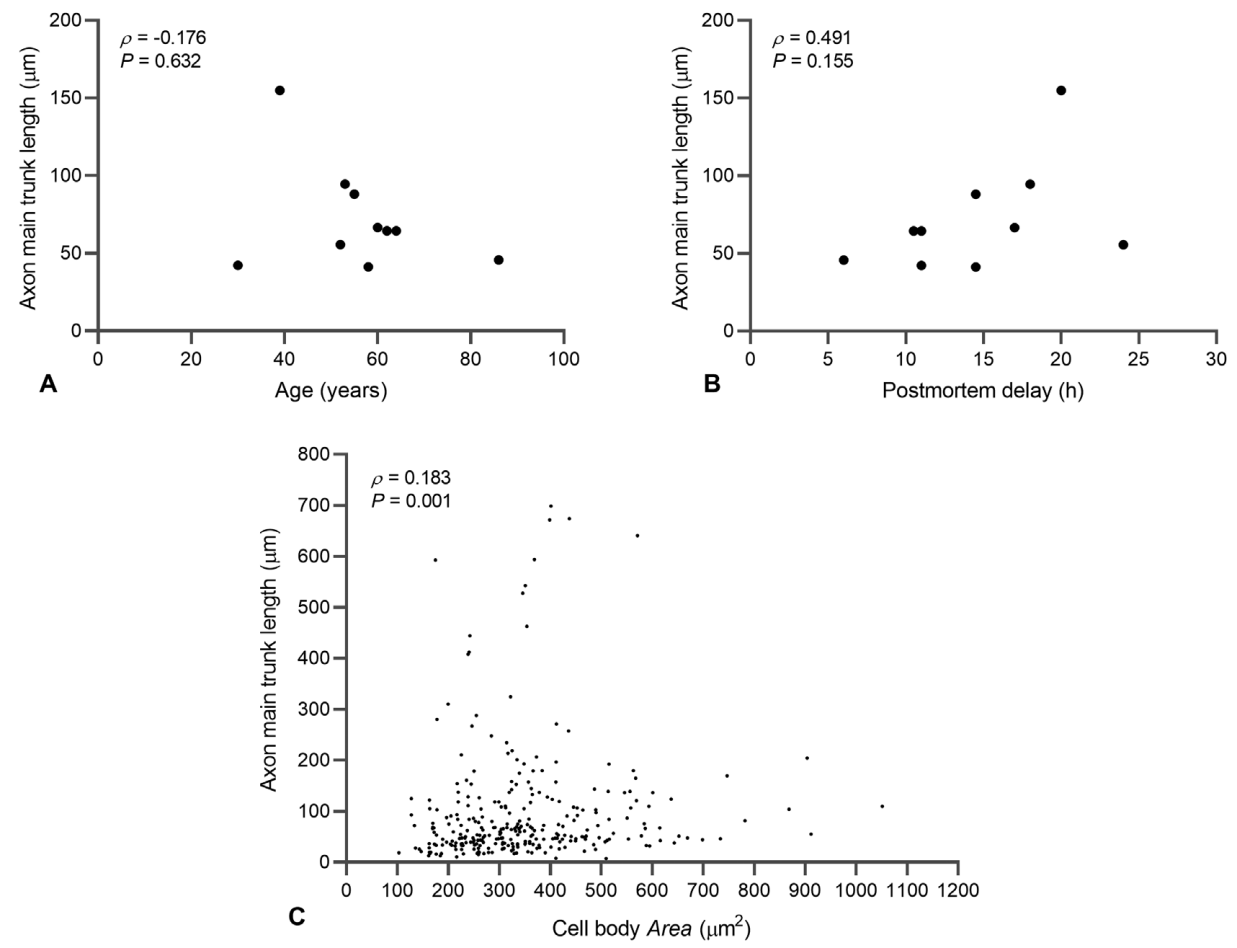
Association between axon main trunk length and possible confounding factors: (**A**) age, (**B**) postmortem delay, and (**C**) cell body *Area*.

There was a weak negative correlation between age and axon main trunk length (ρ = - 0.176), which was not significant (*P* = 0.632) (Figure 7A) and a medium correlation between postmortem delay and axon main trunk length (ρ = 0.491), which was also not significant (*P* = 0.155) (Figure 7B). The correlation between cell body *Area* and axon main trunk length was weak (ρ = 0.183) but significant (*P* = 0.001) (Figure 7C). There were no significant differences in the age of the subjects (*P* > 0.999) and postmortem delay (*P* = 0.476).

## DISCUSSION

In this study, the staining of the axon main trunk was on average significantly longer than in the control group. This was supported by the results of the GLMM analysis, which suggest that the differences between the two groups could be mainly attributed to the differences in neuropathology.

The rapid Golgi method stains the axon up to the beginning of the myelin sheath (25,26), and therefore the length of axon impregnation is expected to be negatively correlated with the myelination level. The axon impregnation length reflects lifespan changes in the myelination process. In infants, when the myelination process is still ongoing (26,45,46), a large part of the axon is visualized on rapid Golgi slides. As myelination increases through childhood, adolescence, and early adulthood, a smaller portion of the axon is visible (47,48). In our study, the beginning of the myelin sheath in the schizophrenia group was, on average, located more distally than in controls. Our findings suggest that in schizophrenia the myelination process is altered, which could be explained by a loss of oligodendrocytes, impaired function of oligodendrocytes in myelin sheath production, or a combination of these factors. Various studies have confirmed that in schizophrenia the absolute number of oligodendrocytes was significantly decreased in Brodmann areas 9 and 24 and in the anterior thalamic nucleus (49-51). Other studies have also found reduced expression of genes associated with oligodendrocytes and myelin (52). There are also direct observations of oligodendrocyte aberrations, which include alterations in the number, spacing, and morphology of oligodendrocytes as well as abnormalities in myelin formation (53). It is speculated that oligodendrocyte abnormalities and consequent myelin dysfunction could alter synaptic function and information processing, which could be important contributing factors for the development of schizophrenia (52).

Based on the neurodevelopmental schizophrenia model, structural lesions in the perinatal period can interact with the process of synaptic pruning in associative circuitry during late adolescence and early adulthood (54-57). It is, therefore, possible that decreased myelination could be related to a disrupted development.

Our findings suggest that not all projection neurons are affected by demyelination equally. On average, there was a clear overall shift toward a greater axon impregnation length in schizophrenia subjects, and the average values of all schizophrenia subjects were higher than those of the control group. In schizophrenia subjects, at least half of the neurons had an axon impregnation length of over 100 μm, whereas in control subjects such length was present in only 10%-20% of neurons. In addition, four schizophrenia subjects had several neurons with a substantially greater axon impregnation length, while this was observed in only one control subject. It is worth noting that the variability in axon staining within a single brain was quite high and was particularly pronounced in subjects CO195 and CO246. These subjects also had the highest mean values in the schizophrenia and control groups, respectively. Our data strongly suggest an overall slight decrease in the level of axon myelination of pyramidal neurons in schizophrenia, which appears to highly affect only a certain proportion of neurons. Neurons with a high level of demyelination may have important functional alteration. Due to the central integrative role of deep layer III neurons in the cortico-cortical network and of large layer V neurons in guiding cortico-striatal executive actions, even demyelination affecting a small proportion of these neurons could cause global changes in cortico-cortical and cortico-subcortical processing (11,12). In this study, we observed no discernible differences in axon staining between layer III and layer V pyramidal neurons, which is particularly interesting since layers III and V have vastly different connections. This suggests that the observed increase in the length of axon staining in schizophrenia subjects may be due to a process that uniformly affects pyramidal neurons of different layers, or at least the pyramidal neurons of layers III and V.

The analysis of potential confounding factors revealed that the size of the neuron cell body (cell body *Area*) was weakly associated with the length of axon staining. We also demonstrated that the subjects’ age at death and postmortem delay on axon staining were unlikely to contribute to the observed differences between the schizophrenia and control groups. Age-related changes in the PFC have previously been described regarding the dendritic field, dendritic spine density, and synapse density (58), however, changes pertaining to the axon are rarely substantially analyzed. Different studies showed different conclusions regarding the types of pyramidal neurons that significantly regressed with age (58). Our analysis did not suggest any substantial differences in axon structure or axon staining related to age. In addition, no differences in tortuosity, base diameter, or average diameter were observed between the groups, suggesting that there were no major structural abnormalities in the axon main trunk. Furthermore, no changes in axon orientation were observed between the schizophrenia and control group.

Due to the limitations of the rapid Golgi method (36,39) and the fact that present observations provide only indirect proof of altered myelination, further research is needed to assess the underlying mechanisms of oligodendrocyte pathology in schizophrenia. Notwithstanding the exact underlying mechanisms, decreased myelination of the axon could alter axon potential propagation and may increase the energy demands of certain neuron types, such as fast-spiking cells (25). In particular, the loss of proximal axon myelination may modify axon potential triggering, which could, change the firing rate of affected neurons. Such a decrease in axon myelination is in line with both the disconnection hypothesis and the two-hit model of schizophrenia as a neurodevelopmental disease (1,9,10).

In conclusion, neurodevelopmental alterations during infancy and early childhood could present an opportunity for early detection of subjects at risk for developing schizophrenia (59). Therefore, decreased myelination could be used as a biomarker for schizophrenia before the onset of the first typical symptoms.
